# UHPLC-HESI-OT-MS-MS Biomolecules Profiling, Antioxidant and Antibacterial Activity of the “Orange-Yellow Resin” from *Zuccagnia punctata* Cav.

**DOI:** 10.3390/antiox9020123

**Published:** 2020-02-01

**Authors:** Jessica Gómez, Mario J. Simirgiotis, Sofía Manrique, Beatriz Lima, Jorge Bórquez, Gabriela E. Feresin, Alejandro Tapia

**Affiliations:** 1 Instituto de Biotecnología-Instituto de Ciencias Básicas, Universidad Nacional de San Juan, Av. Libertador General San Martín 1109 (O), San Juan CP 5400, Argentina; jesicagomez674@gmail.com (J.G.); manriquesofia2@gmail.com (S.M.); blima@unsj.edu.ar (B.L.); gferesin@unsj.edu.ar (G.E.F.); 2 CONICET (Consejo Nacional de Ciencia y Tecnología), CABA, Buenos Aires C1405DJR, Argentina; 3 Instituto de Farmacia, Facultad de Ciencias, Universidad Austral de Chile, Campus Isla Teja, Valdivia 5090000, Chile; 4 Center for Interdisciplinary Studies on the Nervous System (CISNe), Universidad Austral de Chile, Valdivia 5090000, Chile; 5 Laboratorio de Productos Naturales Depto. de Química, Facultad de Ciencias, Universidad de Antofagasta, Av. Coloso S-N, Antofagasta 1240000, Chile; jorge.borquez@uantof.cl

**Keywords:** xanthene’s derivatives, trichothecene, dipping in dichloromethane, biomolecules antioxidants, vedelianin derivatives

## Abstract

This research was designed to investigate the metabolite profiling, phenolics, and flavonoids content as well as the potential antioxidant and antibacterial, properties of orange-yellow resin from *Zuccagnia punctata* Cav (ZpRe). Metabolite profiling was obtained by a ultrahigh resolution liquid chromatography orbitrap MS analysis (UHPLC-ESI-OT-MS-MS). The antioxidant properties were screened by four methods: 2,2-diphenyl-1-picrylhydrazyl assay (DPPH), trolox equivalent antioxidant activity assay (TEAC), ferric-reducing antioxidant power assay (FRAP), and lipid peroxidation in erythrocytes (LP)). The antibacterial activity was evaluated according to the Clinical and Laboratory Standards Institute (CLSI) rules. The resin displayed a strong DPPH scavenging activity (IC_50_ = 25.72 µg/mL) and showed a percentage of inhibition of LP close to that of the reference compound catechin (70% at 100 µg ZpRe/mL), while a moderated effect was observed in the FRAP and TEAC assays. The resin showed a content of phenolic and flavonoid compounds of 391 mg GAE/g and 313 mg EQ/g respectively. Fifty phenolics compounds were identified by ultrahigh resolution liquid chromatography orbitrap MS analysis (UHPLC-PDA-OT-MS) analysis. Thirty-one compounds are reported for the first time, updating the knowledge on the chemical profile of this species. The importance of the biomolecules identified support traditional use of this endemic plant. Furthermore, additional pharmacological data is presented that increase the potential interest of this plant for industrial sustainable applications.

## 1. Introduction

The resins are nonvolatile products of plants, which include surface resins, naturally secreted by plants or internal resins, which can be obtained or collected from incisions. Their chemical composition includes flavonoids, terpenoids, and fatty substances that in some cases are protective barrier for the plant against the attack of some herbivores and other insects [1]. A limited number of families including Fabaceae, Burseraceae, and Pinaceae stand out for their high resin production [1]. Argentina’s Andean region is the habitat of arid and semiarid land species belonging to the genera *Larrea*, *Zuccagnia*, and *Bulnesia* recognized for their high production of resins or exudates, of which there is a lack of knowledge about their potential as sources of biomolecules of pharmacological and industrial interest. Chemical studies carried out in the last decades have reported a limited number of metabolites. Plants exudates are known to possess several biological activities including antimicrobial, antioxidant, anthelmintic, and nematicidal [1,2]. *Zuccagnia punctata* is used in Argentina, to treat injuries and bruises, as a disinfectant of wounds, a repellent of insects, for roof construction in rural areas, and as a vegetable fuel for cooking food. Medicinal plants form a primary means for treatment of various diseases in many parts of the world. In Argentine *Z. punctata* is “the medicinal plant”, it has the largest number of studies of chemistry and biological activity, including antimicrobial, antioxidant, anti-mutagenic, anti-inflammatory, and cytoprotective activities [3].

Principally their bioactivities evidenced have been associated on the basis of the major component (chalcones) [4,5,6,7,8,9].Recently, a selective and reliable characterization of the botanical phenolic profile of *Z.punctata* collected in the northwestern regions of Argentina by liquid chromatography coupled to diode array detector and quadrupole-time of flight mass spectrometry (LC-DAD-Q-TOF-MS) system was reported, highlighting the identification of the major constituent of an ethanolic extract as 4′-hydroxy-2′methoxydihydrochalcone together with other chalcones, flavanones, and caffeic acid derivatives [10].

The interest on the Andean plants is remarkable, because they represent a source very little explored that can offer extracts or biomolecules promising for the study and development of new drugs of pharmacological interest.

In the last decades, several extracts, decoctions, and infusions of medicinal plants and fruits native to Argentina and Chile have been analyzed using the more accurate and reliable quadrupole orbitrap spectrometer (Q-OT-MS), updating significantly the chemical composition in most of the species reported [11,12,13,14,15,16,17,18,19].

In this work, the antioxidant and antibacterial effects complemented with the exhaustive polyphenolic profile of *Zuccagnia punctata* resin are reported, showing the presence of unique bioactive molecules of pharmacological and industrial interest.

## 2. Materials and Methods

### 2.1. Chemicals

Ultra-pure water (<5 µg/L TOC, (Total Organic Carbon) was obtained from a water purification system Arium 126 61316-RO, plus an Arium 611 UV unit (Sartorius, Goettingen, Germany). Methanol (HPLC grade) and formic acid (puriss. p.a. for mass spectrometry) from J.T. Baker (Phillipsburg, NJ, USA) were obtained. Folin-Ciocalteu (FC) reagent, 2,2-diphenyl-1-picrylhydrazyl (DPPH), ferric chloride hexahydrate, 2,4,6-tris(2-pyridyl)-s-triazine, trolox, quercetin, gallic acid, DMSO, and HPLC standards (caffeic acid phenethyl ether (CAPE), galangin, morusin, naringenin, pinocembrin, rhamnetin and shikonin, with purity higher than 95% by HPLC) were purchased from Sigma-Aldrich Chem. Co. (St. Louis, MO, USA) or Extrasynthèse (Genay, France). Cefotaxime was from Argentia^®^ (Bristol-Myers Squibb, Buenos Aires, Argentina). Mueller-Hinton broth was provided by Laboratorio Britania (Buenos Aires, Argentina).

### 2.2. Plant Material

The aerial parts of *Zuccagnia punctata* Cav. (Fabaceae, Caesalpinoideae) were collected in February 2015, on Iglesia district, province of San Juan (Argentina) at an altitude of 1800 m above sea level. A voucher specimen has been deposited at the herbarium of the Botanic Museum of Córdoba (CORD 1125), Universidad Nacional de Córdoba, Argentina.

### 2.3. Z. punctata Orange-Yellow Resin (ZpRe)

The orange-yellow resin was obtained by dipping fresh aerial parts (500 g; 4L of dichloromethane grade HPLC, 1 min; the extraction procedure was done three times), filtered and evaporated under reduced pressure to yield a semisolid yellow-orange resin (10 ± 1% yield *w*/*w*). The ZpRe was stored in a freezer at −40 °C until its use to bioassays, phenolics, and flavonoids identification/quantification as well as in ultrahigh resolution liquid chromatography orbitrap MS analysis (UHPLC-PDA-OT-MS) analysis.

The main chalcones of *Z.punctata*: 2′,4′-dihydroxychalcone, 2′,4′-dihydroxy-3′-methoxychalcone and caffeic acid derivatives, 1-methyl-3-(3′,4′-dihydroxyphenyl)-propyl caffeic acid ester and 1-methyl-3-(4′-hydroxyphenil)-propyl caffeic acid ester were isolated and characterized by analysis of their spectroscopic data (^1^H and ^13^C NMR), which agree with those reported in the literature [3,6].

### 2.4. UHPLC-DAD-MS Instrument

An UHPLC-high-resolution MS machine Thermo Dionex Ultimate 3000 system with PDA detector controlled by Chromeleon 7.3 software (Thermo Fisher Scientific, Waltham, MA, USA) hyphenated with a Thermo Q-Exactive MS focus (Thermo, Bremen, Germany) was used [16].

### 2.5. LC Parameters and MS Parameters

Liquid chromatography was performed using an UHPLC C-18 column (150 × 4.6 mm Acclaim, ID, 2.5 µm; Thermo Fisher Scientific, Bremen, Germany) at 25 °C, hyphenated with a Thermo Q-Exactive MS focus (Thermo, Bremen, Germany) was used. The detection wavelengths were 330,280, 254, and 354 nm, and photodiode array detectors were set from 200–800 nm. Solvent delivery was performed at 1 mL/min using ultra-pure water supplemented with 1% formic acid (A) and acetonitrile with 1% acid formic (B) and a program starting with 5% B at zero time, then maintained 5% B for 5 min, then changing to 30% B within 10 min, then maintaining 30% B for 15 min, then going to 70% B for 5 min, then maintaining 70% B for 10 min, and finally returning to 5% B in 10 min. and keeping this condition for twelve additional minutes to achieve column stabilization before next injection of 20 µL. For the analysis, 5 mg of the resin was dissolved in 2 mL of methanol, filtered through a 200-µm PTFE (polytetrafluoroethylene) filter, and 20 µL was injected in the instrument. Standards and the resin dissolved in methanol were kept at 10 °C during storage in the autosampler. The HESI II and Orbitrap spectrometer (Thermo, Bremen, Germany) parameters were optimized as previously reported [16,17]. Additionally, relevant experimental parameters have been reported recently in detail [18] The Q-Exactive 2.3 SP 2, Xcalibur 2.4 and Trace Finder 3.3 (Thermo Fisher Scientific, Bremen, Germany) were used for UHPLC mass spectrometer control and data processing, respectively.

### 2.6. Total Phenolic (TP) and Flavonoid (F) Content

The total phenolics and flavonoid content of ZpRe was determined by employing total phenols assay by Folin–Ciocalteu reagent and flavonoids by AlCl_3_ assay, both in microplate [18]. The total phenolic was expressed as milligrams of gallic acid equivalents (GAE) per gram of extracts (mg GAE/g ZpRe). Flavonoids were expressed as milligrams of quercetin equivalents (QE) per gram of extracts (mg QE/g ZpRe). The values were obtained using a Multiskan FC Microplate Photometer (Thermo Scientific, Waltham, MA, USA), and are showed as the mean ± standard deviation (SD).

### 2.7. Antioxidant Activity

#### 2.7.1. 2,2-Diphenyl-1-picrylhydrazyl Radical Scavenging Capacity Assay

The Capacity of ZpRe to 2,2-Diphenyl-1-picrylhydrazyl Radical Scavenging (DPPH) was run by the following procedure: DPPH solution (20 mg/L) in methanol was mixed with ZpRe solution at concentrations of 1, 5, 10, 50, and 100 µg/mL [18]. The reaction progress absorbance of the mixture was monitored at 515 nm using a Multiskan FC Microplate Photometer (Thermo Scientific, Waltham, MA, USA). The percentage of the DPPH decoloration was proportional to the five antioxidant concentrations, and the concentration responsible for a decrease in the initial DPPH concentration by 50% was defined and calculated as EC_50_ value, which is showed as the mean ± SD.

#### 2.7.2. Ferric-Reducing Antioxidant Power Assay (FRAP)

The FRAP assay was run in microplate, as previously reported methodology [18,20]. Briefly, FRAP reagent and a methanolic solution of ZpRe resin (1 mg/mL) were mixed; simultaneously, a calibration curve was prepared by mixing FRAP reagent and Trolox solutions, at concentrations between 0 and 1 mmol/L. The absorbance values of mixtures were obtained in a Multiskan FC Microplate Photometer Results were obtained by linear regression from the FRAP-Trolox calibration plot and are showed in equivalent milligrams Trolox/g ZpReresin.

#### 2.7.3. Trolox Equivalent Antioxidant Activity Assay (TEAC)

TEAC assay was run in microplate, as following the previously reported methodology [18,21]. Briefly, a ZpRe methanolic solution was mixed with 200 µL of ABTS, measuring their absorbance at 734 nm after 4 min. Results were obtained by linear regression from a calibration curve constructed with Troloxand are showed expressed as equivalent milligrams Trolox/g ZpRe resin.

#### 2.7.4. Lipid Peroxidation in Erythrocytes

The ability of the ZpReresin at three concentrations (100, 250, and 500 µg/mL) and of catechin at a single concentration (100 µg/mL) to inhibit lipoperoxidation in erythrocytes (LP), induced by tert-Butyl hydroperoxide, was determined. Relevant technical aspects of the trial have been reported recently in detail [15,18]. The values obtained are expressed as percentages of lipid oxidation inhibition.

### 2.8. Antibacterial Activity

#### 2.8.1. Microorganisms

For antibacterial evaluation, were used strains from the American Type Culture Collection (ATCC, Rockville, MD, USA) and clinical isolates from Laboratorio de Microbiología, Hospital Marcial Quiroga, San Juan, Argentina (MQ).The panel comprised the following bacteria: *Staphylococcus aureus* methicillin-sensitive ATCC 29213, *Staphylococcus aureus* methicillin-resistant ATCC 43300, *Staphylococcus aureus* methicillin-resistant-MQ1, *Staphylococcus aureus* methicillin-resistant-MQ2, *Streptococcus agalactiae*-MQ3, *Streptococcus pyogenes*-MQ4, and *Escherichia coli* ATCC 25922.

#### 2.8.2. Antibacterial Susceptibility Testing

Minimum inhibitory concentration (MIC) of ZpRe and antibiotic Cefotaxime (Argentia^®^, Buenos Aires, Argentina) was carried outby broth microdilution techniques, in according to CLSI [22]. The ZpRe was tested from 0.98 to 3000 µg/mL.using an inoculum of each bacterium adjusted to 5 × 10^5^ cells with colony forming units (CFU)/mL. The absorbances at 620 nm were determined in a Multiskan FC Microplate Photometer (Thermo Scientific, Waltham, MA, USA).

### 2.9. Statistical Analysis

The Duncan’s test from InfoStat edition 2016 software (Universidad Nacional de Córdoba, Argentina) was run to determine potential significant differences (*p* < 0.05) in the carried out assays

## 3. Results

### 3.1. UHPLC-PDA-OT-MS Analysis of the Orange-Yellow Resin From San Juan Province, Argentina

Fifty-one compounds were detected in ZpRe by UHPLC-PDA-OT-MS analysis, combining full mass spectra and MS^n^ experiments, of which fifty were tentatively identified including flavonoids, chalcones, caffeic acid derivatives, coumaric acid esters, naphthoquinone, xanthene’s derivatives, trichocethenes; vedelianin derivatives, and others. Several phenolics compounds from ZpRe were rapidly identified using available standards. Thirty-one not previously reported updated the chemical composition of this species. The identification of unknown phenolic compounds xanthene’s characteristics of this bioactive plant was possible from comprehensive analysis of the full scan mass spectra, base peaks chromatograms, and data-dependent scan experiment, since the orbitrap provided high-resolution and accurate mass product ion spectra from precursor ions that are unknown before and within a single run.

The molecular formula was obtained through high resolution accurate mass analysis (HRAM) and matching with the isotopic pattern. The acquisition of the data in the UHPLC-PDA-OT-MS analysis was developed using electrospray negative mode, because compounds with a phenolic OH lose easily the proton in electrospray ionization, giving very good and diagnostic parent ions and fragments.

**Figure 1:**
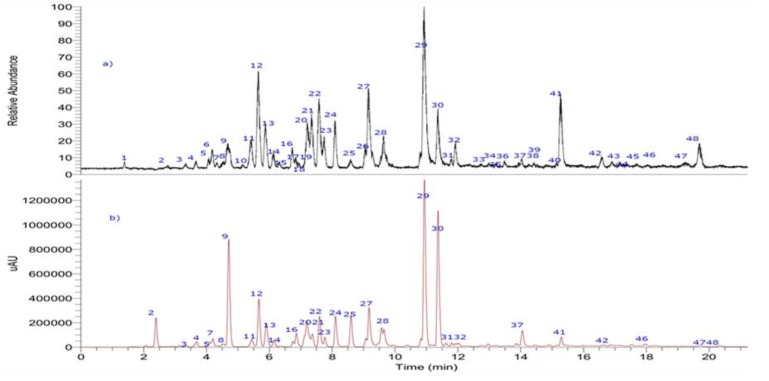
UHPLC-MS (total ion current) chromatograms of ZpRe resin.

**Figure 2:**
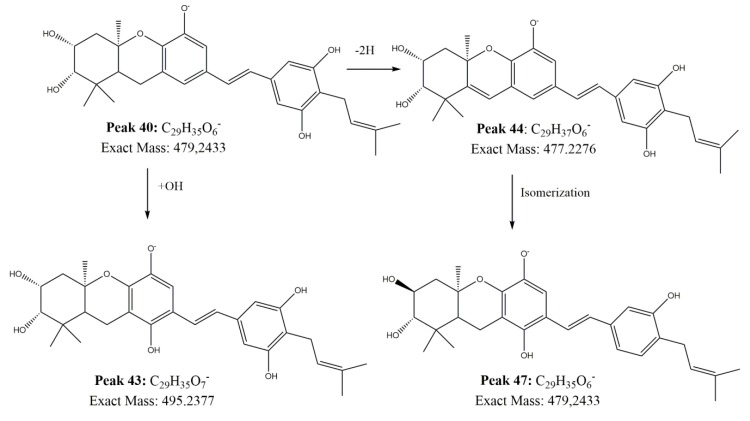
Proposed biosynthesis and structures of Vedelianin and some derivatives in *Z punctata*.

**Figure 3:**
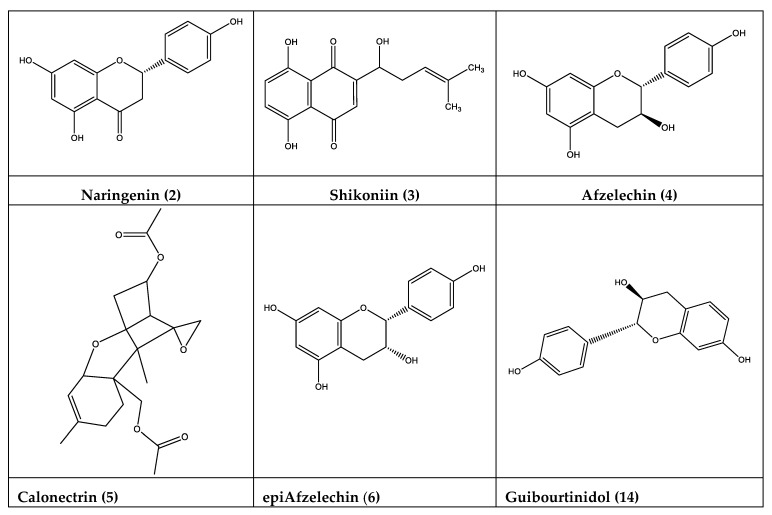

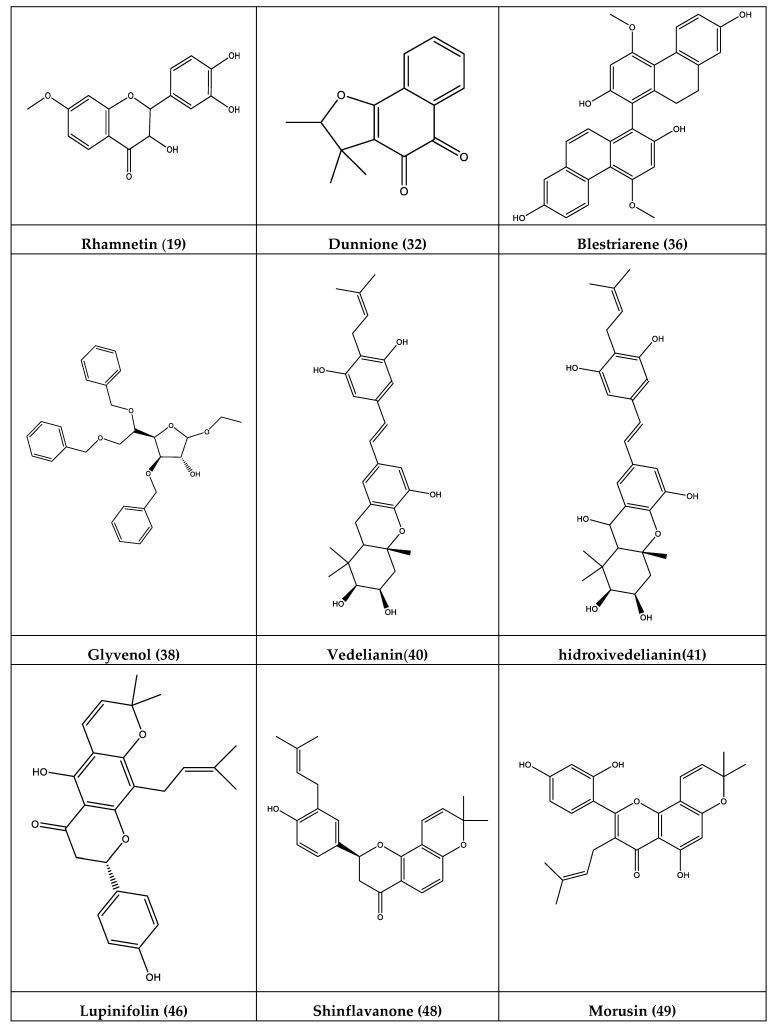
Structures of some newly reported compounds in ZpRe resin.

The metabolome identification of the 50 tentatively identified compounds is developed below, highlighting the relevant information of the 31 compounds that are new reports for the species (See Figure 1, Figure 2 and Figure 3, Table 1, and Supplementary Material Figure S1 for some representative compounds and spectra S1 and S2).

#### 3.1.1. Flavonoids

Peak **2** with a [M − H]^−^ ion at *m*/*z*: 271.06010 was identified as naringenin (C_15_H_11_O_5_^−^) [23], peak **3** as shikoniin (C_9_H_7_O_4_^−^; *m*/*z*: 287.09238) [24]; peak **4** as afzelechin (C_15_H_13_O_5_^−^; *m*/*z*: 273.07575) [25]; peak **6** as epiafzelechin (C_15_H_13_O_5_^−^; *m*/*z*: 273.07660) [26]; peak **7** showing a [M − H]^−^ ion at *m*/*z*: 271.06110 was tentatively identified as an naringenin enantiomer (C_15_H_11_O_5_^−^); peak **8** was identified as 3,7-dihydroxiflavanone [10]; Peak **9** was tentatively identified as 7,8-dihydroxiflavone [10]; peak **10** was proposed as 5-Hydroxy-4′,7-dimethoxyflavanone [27]; peak **11**, was identified as 3,7,8 trihydroxydihydroflavanone [10]; peak **14** with a [M − H]^−^ ion at *m*/*z*: 271.06010 (C_15_H_13_O_4_^−^)was identified as guibourtinidol (2R,3S)-4′,7-Dihydroxyflavan-3-ol), (C_15_H_13_O_4_^−^) [28]; peak **17** was assigned to 7,4′-dihydroxy-5methoxy flavanone [10]; peak **18** is agree to dihydroxyflavanone [10]; peak **19** with a [M − H]^−^ ion at *m*/*z*: 315.0511 was identified as rhamnetin (C_16_H_11_O_7_^−^) [29]; peak **20** was identified as 3,7-dihydroxyflavone [10]; peak **24** and **26** were identified as dihydroxyflavanone pinocembrin [3,10]; and its pinocembrin isomer; showing both compounds similarly MS/MS compared with authentically reference compounds; peak **27** was identified as 3,5,7-trihydroxyflavone (galangin) compared with compound of reference standard [3]; peak **33** was tentatively proposed as flavanone supported by UV signal at 287nm and MS/MS fragmentation (C_15_H_11_O_3_^−^; *m*/*z*: 239.07097); Peak **48** with a [M − H]^−^ ion at *m*/*z*: 389.17566 was identified as shinflavanone (C_25_H_25_O_4_^−^; UV signals 287 nm) [30]; while peak **49** with a [M − H]^−^ ion at *m*/*z*: 419.1502 was identified as prenylated flavonoid morusin (C_25_H_23_O_6_^−^) [31]; peak **50** was tentatively identified as 8-C-Prenyl-6″,6″-dimethylpyrano [2″,3″:7,6] flavanone (C_25_H_25_O_3_^−^; *m*/*z*: 373.18060); peak **51** with [M − H]^−^ ion at *m*/*z*: 389.17572 and UV signals (287 nm) was identified as 3,7-dihydroxyflavone (C_25_H_25_O_4_).

#### 3.1.2. Chalcones

Peaks **25** with a [M − H]^−^ ion at *m*/*z*: 253.08669 was identified as 2′-hydroxy-4-methoxychalcone (C_16_H_13_O_3_^−^), while peaks **34**, and **35** were identified as characteristics chalcones reported to *Z. punctata* 2′,4′-dihydroxychalcone and 2′,4′-dihydroxy-3′-methoxychalcone; both chalcones were determined by MS/MS experiment and compared with authentic reference compounds previously isolated [3].

#### 3.1.3. Caffeic Acid Derivatives

Peak **12** was identified as 1-methyl-3-(3′,4′-dihydroxyphenil)-propyl caffeic acid ester by their MS/MS properties compared with authentic reference compounds previously isolated [7,10]; while peaks **13** and **15** with a [M − H]^−^ ion at *m*/*z*: 343.11838 and 343.11870 respectively, were tentatively identified as 1-methyl-3-(3′,4′-dihydroxyphenil)-propyl caffeic acid ester isomers (C_19_H_19_O_6_^−^); peaks **21** was identified as 1-methyl-3-(4′-hydroxyphenil)-propyl caffeic acid ester by their MS/MS compared with authentically reference compounds previously isolated [7,10]; in the same way, peak **22** was assigned to 2-methyl-3-(3-hydroxy-4′-methoxyphenyl)-propyl caffeic acid ester, while peak **23** is supported by its mass properties as an isomer of compound **21**; peak **28** was tentatively identified as recognized caffeic acid phenetyl ether; peak **42** was identified as 3,7-dimethyl-2,6-octadienyl caffeic acid ester; and peak **45** was identified as 3,7-dimethyl-2,6-octadienyl caffeic acid ester.

#### 3.1.4. Coumaric Acid Esters

Peak **29** was identified as 4′-terbutyloxyphenyl *p*-coumaric acid ester [10], while peak **30** was tentatively proposed to 1-methyl-3-(4′-hydroxyphenyl)-propyl *p*-coumaric acid ester [10]; peak **31** by identical MS/MS properties and UV signals (C_19_H_19_O_4_^−^; *m*/*z*: 311.1289; 231–308–347 nm) was identified as a isomer of compound **30**; and peak **37** was proposed supported by MS/MS fragmentation as isomer of compound **29**.

#### 3.1.5. Xanthene’s Derivatives, Trichothecenes; Vedelianin Derivatives, and Others

Peak **5** with a [M − H]^−^ ion at *m*/*z*: 349.16456 was identified as trichothecene calonectrin (C_19_H_25_O_6_^−^) [32]. Peak **16** with a [M − H]^−^ ion at *m*/*z*: 287.09232was identified as naphthoquinone derivative, shikoniin isomer (C_16_H_15_O_5_^−^).

Peak **32** with a [M − H]^−^ion at *m*/*z*: 349.16456 (C_15_H_13_O_3_^−^), was identified as dunnione. Peak **36** was identified as blestriarene B, with a [M − H]^−^ ion at *m*/*z*: 479.14891 (C_30_H_23_O_6_^−^) [33].

Peak **38** with a [M − H]^−^ ion at *m*/*z*: 477.22717 (C_29_H_33_O_6_^−^) was identified as glyvenol (tribenoside).

Peaks **40**, **41**, **43**, **44**, and **47** are structurally related, were thus assigned based on their mass properties and characteristic UV signals as the hexahydroxanthene derivative vedelianin (peak 40; C_29_H_35_O_6_^−^; *m*/*z*: 479.24338) [34] and some of its derivatives as follows: Peak **41** with a [M − H]^−^ ion at *m*/*z*: 495.23813 (C_29_H_35_O_7_^−^)was identified as hidroxivedelianin; peak **43** with a [M − H]^−^ ion at *m*/*z*: 495.23773 (C_29_H_35_O_7_^−^)was tentatively assigned to hidroxivedelianin isomer, peak **44**, was identified as a reduced vedelianin (C_29_H_33_O_6_^−^; *m*/*z*: 477.22769); while peak **47** (C_29_H_35_O_6_^−^; *m*/*z*: 479.24338) was assigned to other vedelianin isomer, supported by identical MS/MS. A proposed biosynthesis and structures of vedelianin and some derivatives in *Z punctata* are showed in Figure 2. Peak **46** was tentatively identified as lupinifolin [35].

### 3.2. Total Phenolic and Flavonoid Contents and Antioxidant, and Antibacterial Activities

The orange-yellow resin from *Z.punctata* (ZpRe) displayed a stronger DPPH scavenging activity with an EIC_50_ 25.72 µg/mL, as well as an outstanding inhibition of lipid peroxidation in erythrocytes (70% percent at 100 µg ZpRe/mL), this was comparable to the value shown by the reference compound catechin (74% at 100µg/mL) (Table 2) and to the values shown recently by others Andean species [14,15,18]. Phenolic antioxidant compounds acting as free radical scavengers can delay or inhibit lipid oxidation processes. This protection of polyunsaturated fatty acids against free radical damage may explain or supports phenolic compounds as a valuable natural product with potential to improve human health [36] Regarding FRAP and ABTS results, the ZpRe exhibited moderate effect in both trials. The ZpRe presented a high content of TP, with values of 391 mg GAE/g ZpRe, where as approximately eighty percent correspond to flavonoids (313 mg QE/g ZpRe).

Results of antibacterial activity are depicted in Table S1 (Supplementary Material).The ZpRe showed activity against Gram-positive bacteria, including *Staphylococcus aureus* methicillin-resistant ATCC 43300, *S. aureus* methicillin-resistant-MQ-1, *S. aureus* methicillin-resistant-MQ-2, *S. aureus* methicillin-sensitive ATCC 25923 and *Streptococcus pyogenes* (MICs values were between 125 and 250 µg/mL). However, the ZpRe resin was not active against most of the other strains tested (MIC values >250 µg/mL).

The antioxidant, antimicrobial, and other biological activities have been associated, by several authors, with the content of flavonoids and chalcones and some specific flavonoids such as pinocembrin [3,4,5,6,7,8,9]. In a previous study, the quantification of selected markers performed by HPLC-UV method, showed that the resin contains on average 3.18; 3.20; 16.04; and 12.84 g of pinocembrin (24), galangin (27), 2′,4′-dihydroxychalcone (34) and 2′,4′-dihydroxy-3′methoxychalcone (35) respectively, each quantified in 100 g of ZpRe [8].

However, the full UHPLC-MS identification of thirty one biomolecules for the first time in this species (peaks 2–7, 13–16, 19, 23, 25, 26, 31–33, 36–41, 43, 44, 46–51), some of them showing a broad spectra of pharmacological properties, including antioxidant and antimicrobial, canprovide additional and relevant support for the activities displayed by ZpRe resin. Figure 3 and Figure S2 show the structures of some newly reported compounds in the resin of this plant.

Pharmacological activities of naringenin (**2**), as therapeutic agent to treat different diseases, such as cancer, diabetes, cardiovascular diseases and neurological disorders, oxidative stress and inflammation have been extensively reported [37,38]. Additionally, antibacterial activity against *Salmonella thypi*, *Staphylococcus aureus,* and *Escherichia coli* ATCC as well as their antinociceptive and anti-inflammatory effect in mice model, have been also reported [39,40,41,42]. Furthermore, the free radical scavenging and antioxidant properties havebeen associated with improvements experienced by rats with diabetes type I treated with naringenin [43,44]. The protective effect against metabolic diseases of naringenin is supported by its ability to scavenging some free radicals, by its ability to induce antioxidant enzymes and targeting on phosphoinositide 3-kinase/protein Kinase B/nuclear factors [38]. These mechanisms are involved in the neuroprotective effect recently reported by Chandran et al. [45].

Shikonin (3) have demonstrated a broad spectrum of relevant biological activities such as, antioxidant, anti-inflammatory, antithrombotic, antimicrobial, wound healing effects, as well as neuroprotective effects against cerebral ischemia/reperfusion injury associated or supported to its antioxidant properties [46,47,48,49,50,51]. On the other hand, shikonin inhibited the proliferation of three human pancreatic cancer cell lines, and potentiated synergistically the cytotoxic effect of the gemcitabine a chemotherapeutic drug [52].

Additionally, afzelechin (**4**), epiafzelechin (**6**), and some catechol derivatives have been extensively reported as antioxidant compounds. Moreover, in relation to the antioxidant properties of afzelechin (**4**) its neuroprotective effect related to glutamate-induced neurotoxicity in HT22 cells has been informed [53,54,55,56].

Compound **25** (2-hydroxy-4–methoxychalcone) has been reported as antiangiogenic, antitumoral, glutathione S-transferase inhibitor, and as therapeutic agent to treat atherosclerosis. In addition, several epidemiological studies support the idea that regular consumption of fruits and vegetables rich in flavonoids reduces the risk of cardiovascular diseases [57,58,59].

In the other hand, the strong antibacterial activity of blestriarene B (36) and its antibacterial activity against *Streptococcus mutans* and *Staphylococcus aureus* (with MICs values of 12.5 and 6.25 µg/mL respectively) has been also reported [33].

Vedelianin (**40**) has been recently reported as a potent antiproliferative agent against several cancer cell lines [60,61]. The potential of morusin (**49**) against human colon rectal cancer and human cell lung cancer has been informed by Chang Lee et al. [62] and Park et al. [63]. Also, the cytotoxic activity of lupinifolin (**46**) in the cell line P-388 [35] was reported.

The tribenoside glyvenol, (**38**) was used clinically for hemorrhoidal disease associated with coagulation, inflammation, and wounds. Kikkawa et al. (2010) [64] reported that tribenoside interacts with epidermal cells and regulates the expression and localization of laminins to help reconstruct basement membranes in wound healing of hemorrhoids. Evidence exists to recommend the use of tribenoside as a fast, effective and safe option for the local treatment of low-grade hemorrhoids [65].

Regarding guibourtinidol (**14**), recently, the hepatoprotective activity and powerful antioxidant properties of *Cassia abbreviata* root extract, rich in (epi)-catechin, (epi)-afzelechin, (epi)-guibourtinidol, and (ent)-cassiaflavan monomers as well as their dimers and trimers has been reported [66].

Caffeic acid phenethyl ester (CAPE, **28**) is a bioactive compound of propolis and the exudate extract. It is known that CAPE possesses antioxidant, anti-inflammatory, anticancer, and cytotoxic properties and is a versatile therapeutically active polyphenol and an effective adjuvant of chemotherapy [67,68,69,70,71].

## 4. Conclusions

Fifty phenolics compounds were identified by ultrahigh resolution liquid chromatography orbitrap MS analysis (UHPLC-PDA-OT-MS). Thirty-one are reported for the first time, updating the knowledge of the chemical profile of this species. The importance of the biomolecules identified support its traditional use. Herein, more scientific data on bioactivity and chemistry is showed for this plant that increase its potential for sustainable applications and industrial interest that *Z. punctata* offers, a species that grows in semi-arid Andean areas.

## Figures and Tables

**Table 1:** Resolution UHPLC-PDA-Q-Orbitrap identification of biomolecules from ZpRe resin.

Peak	Retention Time (min)	UV Max	Tentative Identification	Elemental Composition [M − H]^−^	Theoretical Mass (*m*/*z*)	Measured Mass (*m*/*z*)	Accuracy(δ ppm)	MS^n^Ions
**1**	1.33		unknown	C_16_H_15_O_5_	85.00343			
**2**	2.77	279	Naringenin ^a^	C_15_H_11_O_5_	271.06110	271.0601	3.67	
**3**	3.35	279–367	Shikoniin ^a^	C_16_H_15_O_5_	287.09325	287.0923	3.40	
**4**	3.65	279	Afzelechin ^a^	C_15_H_13_O_5_	273.07575	273.0766	3.22	
**5**	4.05	-	Calonectrin ^a^	C_19_H_25_O_6_	349.16456	349.1654	2.58	85.00342
**6**	4.18	279	EpiAfzelechin ^a^	C_15_H_13_O_5_	273.07575	273.0766	3.10	
**7**	4.28	287	Naringenin enantiomer ^a^	C_15_H_11_O_5_	271.06195	271.0611	3.67	151.0394:109.0286
**8**	4.31	287	3,7-dihydroxiflavanone	C_15_H_11_O_4_	255.06519	255.0661	3.67	151.0394:109. 0286
**9**	4.61	287	7,8-dihydroxiflavone	C_15_H_11_O_4_	255.06519	255.0661	3.11	237.0553
**10**	5.12	287	5-hydroxy-4′,7-dimethoxyflavanone	C_17_H_15_O_5_	299.09140	299.0929	2.95	285.0403;179.0345, 135.0444
**11**	5.38	287	3,7,8-trihydroxydihydroflavanone	C_15_H_11_O_5_	271.06010	271.0610	3.33	253.0503;225.0552; 197.0603;151.0029
**12**	5.67	234-292-325	1-methyl-3-(3′,4′-dihydroxyphenyl)-propyl caffeic acid ester	C_19_H_19_O_6_	343.11761	343.1187	2.49	179.0344;161.0236; 135.0443
**13**	5.88	236-277-312	1-methyl-3-(3′,4′-dihydroxyphenyl)-propyl caffeic acid ester isomer ^a^	C_19_H_19_O_6_	343.11761	343.1183	2.22	257.0818;179.0345; 151.0393;135.0444; 107.0494
**14**	5.88	236-277-312	Guibourtinidol ^a^	C_15_H_13_O_4_	257.08084	257.0816	3.10	179.0345;151.0393; 135.0444;107.0494
**15**	6.14	279–367	1-methyl-3-(3′,4′-dihydroxyphenil)-propyl caffeic acid ester isomer ^a^	C_19_H_19_O_6_	343.11853	343.1187	2.66	287.0818;151.0393; 119.0495;107.0494
**16**	6.14	279–367	Shikoniin isomer ^a^	C_16_H_15_O_5_	287.09140	287.0923	3.18	151.0393;119.0495; 107.0494
**17**	6.27	285	7,4′-dihydroxy-5-methoxy-flavanone	C_16_H_13_O_5_	285.07575	285.0766	3.19	149.9952;119.0495
**18**	6.73	287	Dihydroxyflavanone	C_15_H_11_O_4_	255.06519	255.0669	3.05	237.0553;209.0604; 195.0400
**19**	6.91	251–349	Rhamnetin ^a^	C_16_H_11_O_7_	315.04993	315. 0511	3.18	185.0034,146.93796
**20**	7.18	246-324-237	3,7-dihydroxyflavone	C_15_H_9_O_4_	253.05029	253.0495	2.99	208.0524;223.0326; 195.0447; 180.0576
**21**	7.31	277 314	1-methyl-3-(4′-hydroxyphenil)-propyl caffeic acid ester	C_19_H_19_O_5_	327.12357	327.1227	2.64	135.0443
**22**	7.55	242, 291–324	2-methyl-3-(3-hydroxy-4′-methoxyphenyl)-propyl caffeic acid ester	C_20_H_21_O_6_	357.13409	357.1332	2.32	343.1104;193.0500; 179.0343;161.0237;135.0440
**23**	7.70	249-285-323	1-methyl-3-(4′-hydroxyphenil)-propyl caffeic acid ester isomer ^a^	C_19_H_19_O_5_	327.12380	327.1227	3.01	179.0344;163.0394; 135.0443;119.0494
**24**	8.04	235-343	Pinocembrin	C_15_H_11_O_4_	255.06601	255.0651	3.23	227.0907;213.0503; 164.0109;151.0029; 123.0080
**25**	8.54	239–306	2′hydroxy-4-methoxychalcone ^a^	C_16_H_13_O_3_	253.08592	253.0866	3.02	
**26**	9.00	291	Pinocembrin isomer ^a^	C_15_H_11_O_4_	255.06599	255.0651	3.17	227.0709;213.0553; 164.0109;145.0642; 123.0080
**27**	9.14	267-315-360	Galangin(3,5,7-trihydroxyflavone)	C_15_H_9_O_5_	269.04579	269.0453	3.22	213.0551;169.0653
**28**	9.39	242-268-310-357	Caffeic acid phenetyl esther	C_17_H_15_O_14_	283.09649	283.0794	3.38	
**29**	9.61	271	4′-terbutyloxyphenyl *p*-coumaric acid ester	C_19_H_19_O_4_	311.12866	311.1286	2.91	163.0394;119.0490
**30**	9.67	231-308 347	1-methyl-3-(4′-hydroxyphenyl)-propyl *p*-coumaric acid ester	C_19_H_19_O_4_	311.12779	311.1289	2.91	179.0344;163.0394; 134.0366;119.0490
**31**	9.88	231-308-347	1-methyl-3-(4′-hydroxyphenyl)-propyl *p*-coumaric acid ester isomer ^a^	C_19_H_19_O_4_	311.12866	311.1289	2.81	179.0344;163.0394; 135.0444;119.0494
**32**	10.80	277–312	Dunnione ^a^	C_15_H_13_O_3_	241.08592	241.0866	2.98	
**33**	10.88	287	Flavanone *	C_15_H_11_O_3_	239.07027	239.0709	2.91	197.0603;169.0653; 153.0186;135.0080; 121.0280
**34**	10.90	232–346	2′,4′-dihydroxychalcone	C_15_H_11_O_3_	239.07027	239.0710		197.0603;169.0653; 153.0186;135.0080; 121.0280
**35**	11.37	232–345	2′,4′-dihydroxy-3′-methoxychalcone	C_16_H_13_O_4_	269.08167	269.0808	3.08	
**36**	11.77	285	Blestriarene B ^a^	C_30_H_23_O_6_	479.14957	479.14891	1.36	
**37**	11.91	280–323	4′-terbutyloxyphenyl *p*-coumaric acid ester isomer ^a^	C_19_H_19_O_4_	311.12779	311.1286	2.71	179.0344;161.0237; 135.0442
**38**	12.72	283	Glyvenol ^a^	C_29_H_33_O_6_	477.22754	477.2271	0.78	
**39**	12.95	293	1-methyl-3-(3′,4′-dihydroxyphenil)-propyl caffeic acid ester isomer ^a^	C_19_H_19_O_6_	343.11761	343.1183		179.0344; 161.0238; 135.0444: 109.0286
**40**	13.19	280	Vedelianin ^a^	C_29_H_35_O_6_	479.24882	479.2433	1.17	
**41**	13.47	280	Hidroxivedelianin ^a^	C_29_H_35_O_7_	495.23773	495.2381	0.82	161.0238;135.0443; 109.0286
**42**	13.93		3,7-dimethyl-2-octaenyl caffeic acid ester	C_19_H_21_O_4_	313.14344	313.1443	2.91	
**43**	14.04	267–357	hidroxivedelianin isomer ^a^	C_29_H_35_O_7_	495.23785	495.2377	0.25	479.2432;239.0710; 179.0345;161.0238; 135.0442
**44**	14.42	285	Vedelianin reduced ^a^	C_29_H_33_O_6_	477.22717	477.2276	1.10	
**45**	15.26	285–320	3,7-dimethyl-2,6-octadienyl caffeic acid ester (geranyl Caffeate)	C_19_H_23_O_4_	315.16993	315.1600	3.10	178.0265;134.0364; 133. 0289
**46**	16.58	289-320	Lupinifolin	C_25_H_25_O_6_	405.16965	405.1754	−1.27	
**47**	16.58	289	Vedelianin isomer ^a^	C_29_H_35_O_6_	479.24882	479.2433	1.17	
**48**	16.89	287	Shinflavanone ^a^	C_25_H_25_O_4_	389.17474	389.1756	2.37	371.1654
**49**	17.39	285	Morusin ^a^	C_25_H_23_O_6_	419.1502	419.14891	2.21	363.0873;179.0344; 151.0354;109.0286
**50**	19.23	291	8-C-Prenyl-6″,6″-dimethylpyrano [2″,3″:7,6] flavanone ^a^	C_25_H_25_O_3_	373.19782	373.1806	2.09	
**51**	19.68	287	Shinflavanone isomer ^a^	C_25_H_25_O_4_	389.17474	389.1757	2.52	

^a^ New reports for the species *Zuccagnia punctata*.

**Table 2:** Antioxidant assays and total phenolic and flavonoids content of ZpRe from *Z. punctata*.

**Phenolics Content**	**ZpRe**
Total phenolics (mg GAE/g ZpRe)	391.40 ± 2.18
Flavonoids (mg QE/g ZpRe)	313.18 ± 3.10
**Antioxidant Assay**	
DPPH (EC_50_ in µg ZpRe/mL)	25.72 ± 1.51
FRAP (mg TE/g ZpRe)	1.74 ± 0.13
TEAC (mg TE/g ZpRe)	1.25 ± 0.01
Percentage LP (at 100 µg ZpRe/mL)	70.14 ± 2.26
Percentage LP (at 100 µg catechin/mL)	74.14 ± 1.25

No significant differences were found between the three samples. ANOVA (analysis of variance) followed by Dunett’s comparison test was used (significance *p* < 0.05). DPPH: (2,2-diphenyl-1-picrylhydrazyl; TEAC: trolox equivalent antioxidant activity assay; FRAP: ferric-reducing antioxidant power assay.

## Supplementary Materials

The following are available online at https://www.mdpi.com/2076-3921/9/2/123/s1. Figure S1: (a–h). Full orbitrap MS spectra and structures of representative compounds 3, 4, 14, 28, 30, 40, 43, and 48. Figure S2: Structures of newly reported compounds to ZpRe. Table S1. Antimicrobial activity of ZpRe.
