# Supplementary material for: UHPLC-HESI-OT-MS-MS Biomolecules Profiling, Antioxidant and Antibacterial Activity of the “Orange-Yellow Resin” from Zuccagnia punctata Cav
Source: Antioxidants (Basel). 2020 Feb 1;9(2):123. doi: 10.3390/antiox9020123 (PMC7070614; doi:10.3390/antiox9020123)
Supplement: Supplementary file 1 [file antioxidants-09-00123-s001.pdf]

Suplementary material

Article

UHPLC-HESI-OT-MS-MS biomolecules profiling, antioxidant andantibacterial activity of the “orange-yellow resin “from *Zuccagnia punctate* Cav.

Jessica Gómez[1, 2], Mario J. Simirgiotis[3, 4 *], Sofía Manrique[1,2], Beatriz Lima [1,2], Jorge Bórquez[5], Gabriela E. Feresin[1,2] and Alejandro Tapia [1*]

[1] Instituto de Biotecnología-Instituto de Ciencias Básicas, Universidad Nacional de San Juan, Av. Libertador General San Martín 1109 (O), San Juan CP 5400, Argentina; jesicagomez674@gmail.com (J.G.);; gferesin@unsj.edu.ar

[2] CONICET (Consejo Nacional de Ciencia y Tecnología), CABA, Buenos Aires C1405DJR, Argentina;

[3] Instituto de Farmacia, Facultad de Ciencias, Universidad Austral de Chile, Campus Isla Teja, Valdivia 5090000, Chile

[4] Center for Interdisciplinary Studies on the Nervous System (CISNe), Universidad Austral de Chile, Valdivia 5090000, Chile

[5] Laboratorio de Productos Naturales Depto. de Química, Facultad de Ciencias, Universidad de Antofagasta, Av. Coloso S-N, Antofagasta 1240000, Chile; jorge.borquez@uantof.cl

*Correspondence: mario.simirgiotis@uach.cl (M.J.S.); atapia@unsj.edu.ar (A.T.);
Tel.: +56-063-2244369 (M.J.S.); +54-264-4211700-294 (A.T.)
Received: date; Accepted: date; Published: date

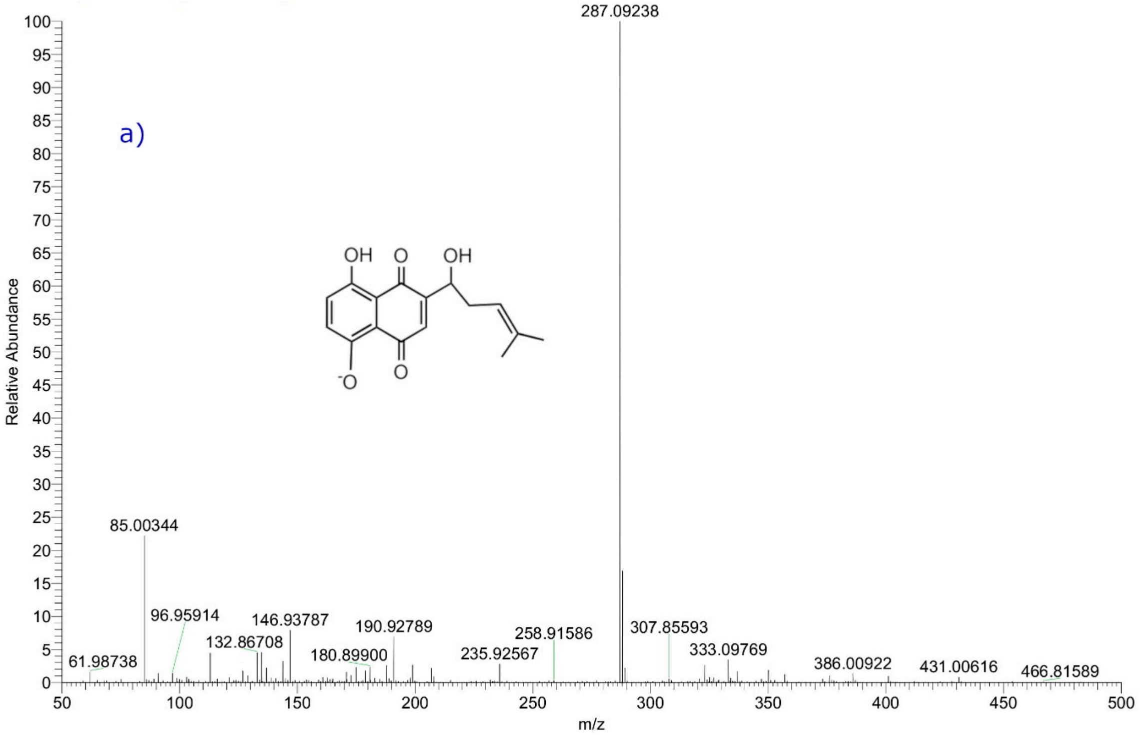
287.09238
a)
Relative Abundance
85.00344
96.95914
132.86708
146.93787
190.92789
180.89900
61.98738
235.92567
258.91586
307.85593
333.09769
386.00922
431.00616
466.81589
m/z

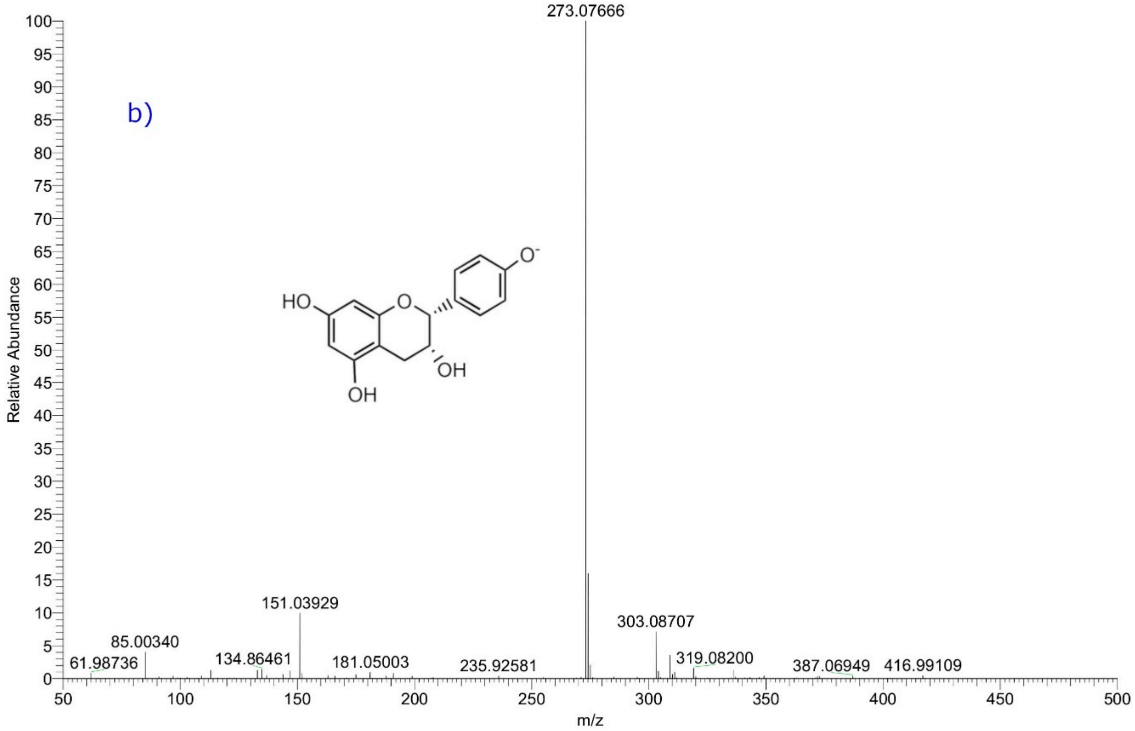
273.07666
b)
Relative Abundance
151.03929
85.00340
61.98736
134.86461
181.05003
235.92581
303.08707
319.08200
387.06949
416.99109
m/z

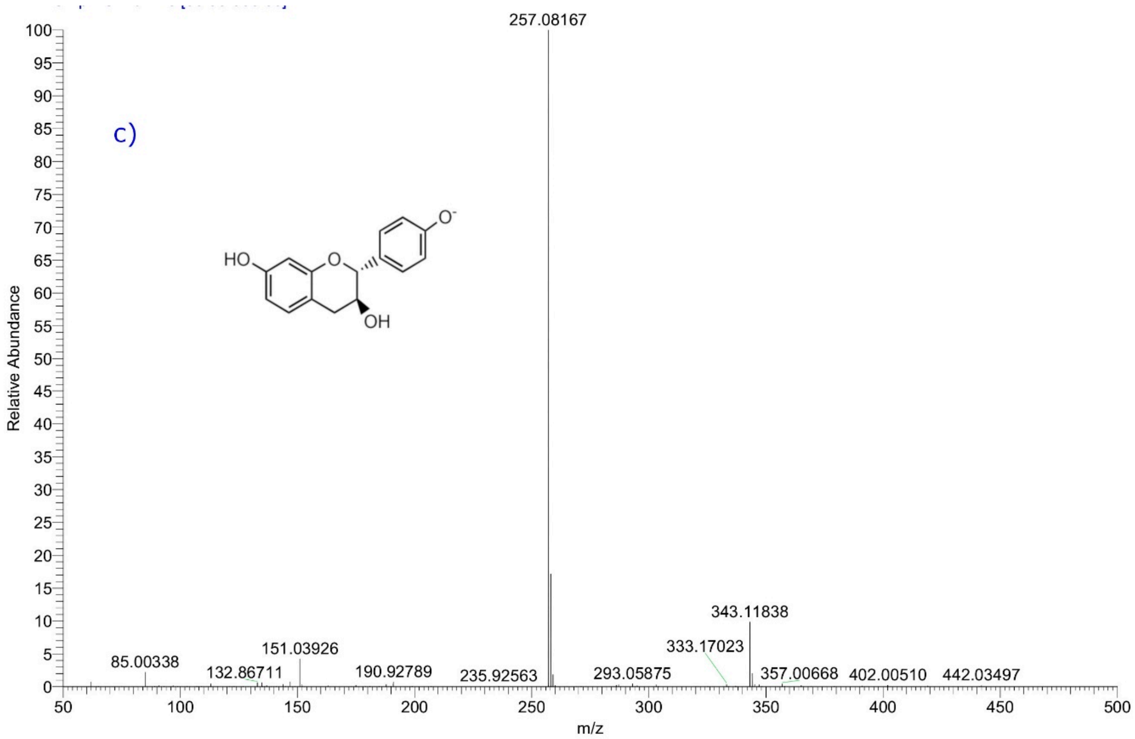
c)
257.08167
343.11838
333.17023
151.03926
85.00338
132.86711
190.92789
235.92563
293.05875
357.00668
402.00510
442.03497
HO
O
O-
OH
Relative Abundance
m/z

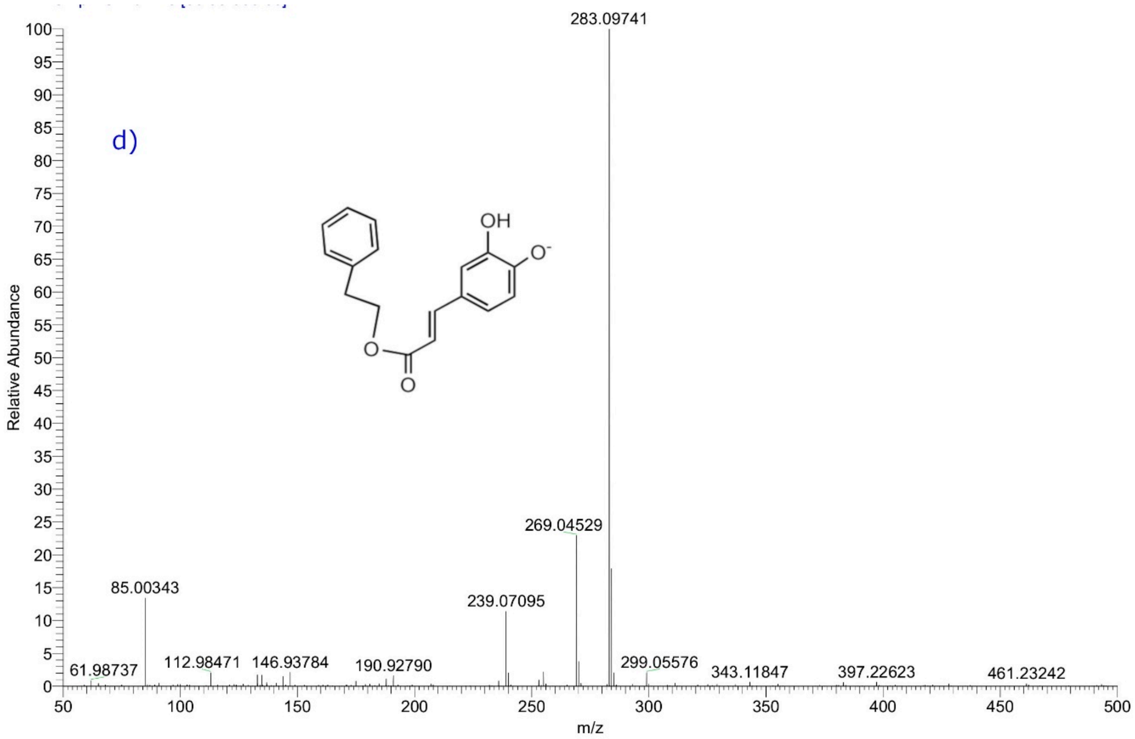
d)
283.09741
269.04529
239.07095
85.00343
61.98737
112.98471
146.93784
190.92790
299.05576
343.11847
397.22623
461.23242
OH
O-
O
O
Relative Abundance
m/z

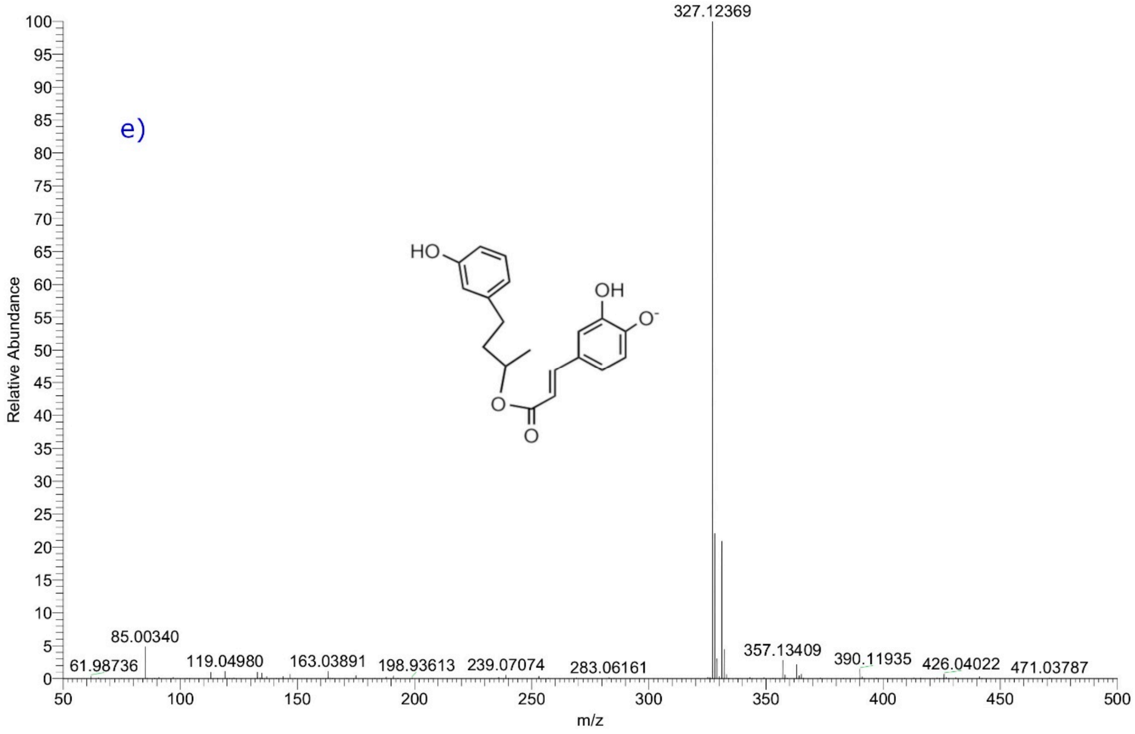
e)
327.12369
85.00340
61.98736
119.04980
163.03891
198.93613
239.07074
283.06161
357.13409
390.11935
426.04022
471.03787
HO
OH
O
O
O
Relative Abundance
m/z

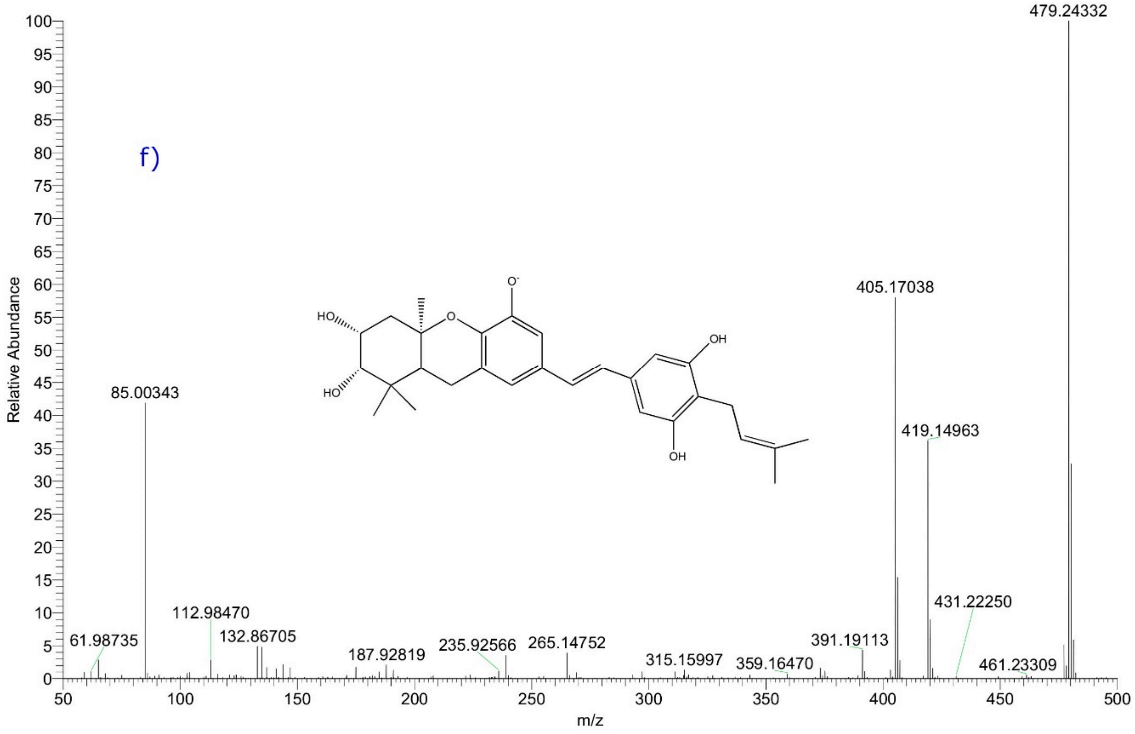
f)
479.24332
405.17038
419.14963
85.00343
431.22250
112.98470
61.98735
132.86705
187.92819
235.92566
265.14752
315.15997
359.16470
391.19113
461.23309
HO
HO
O
OH
OH
Relative Abundance
m/z

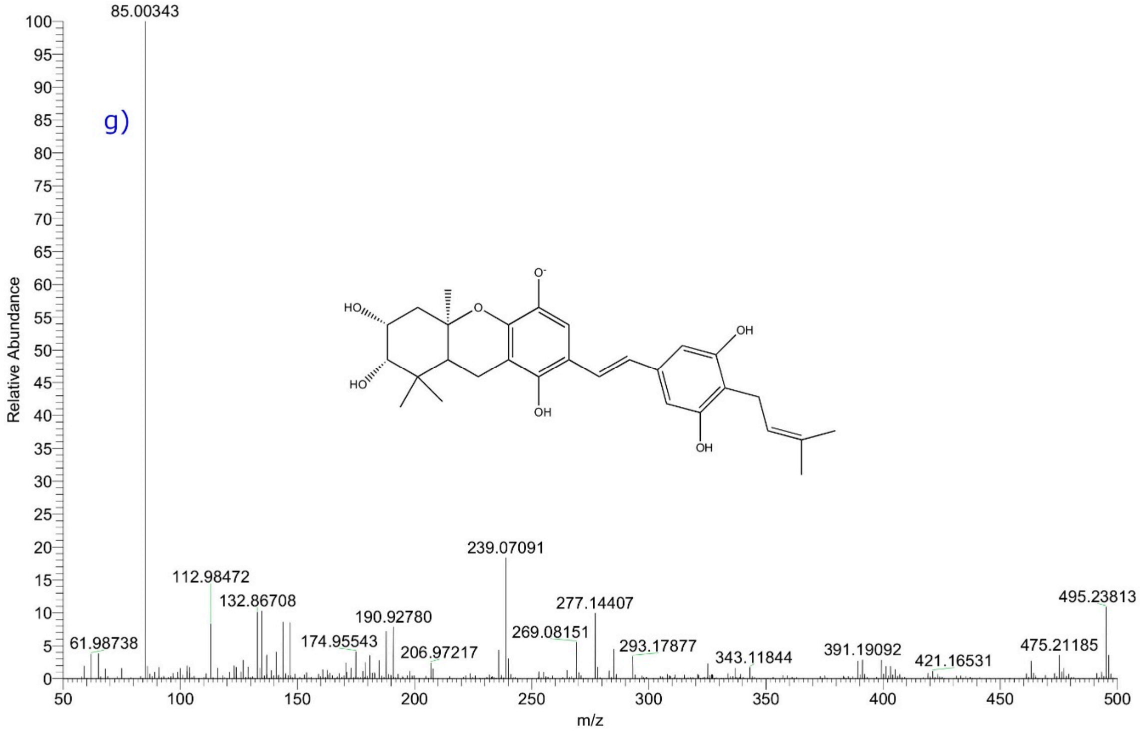

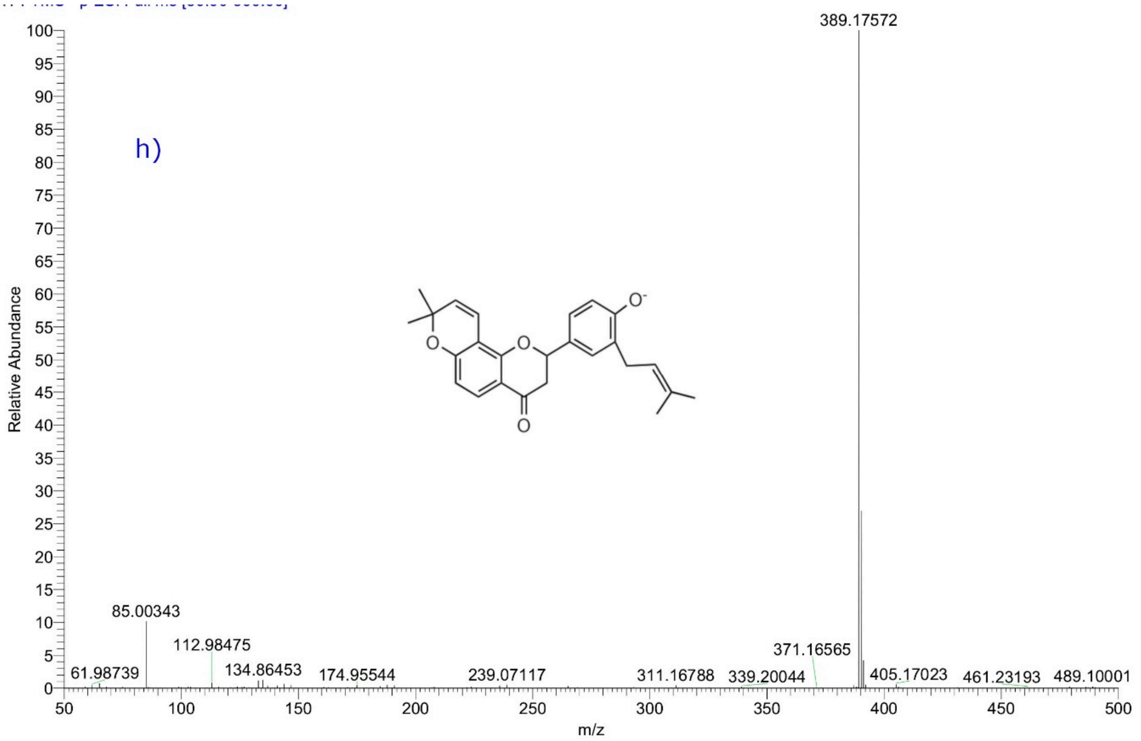

Figure S1. (a–h). Full orbitrap MS spectra and structures of representative compounds 3, 4, 14, 28, 30, 40, 43 and 48.

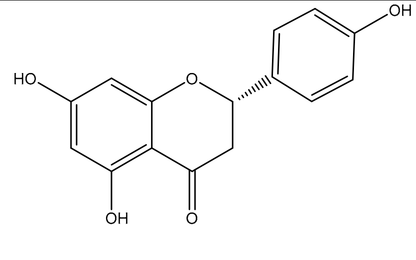	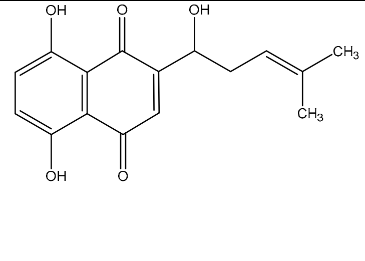	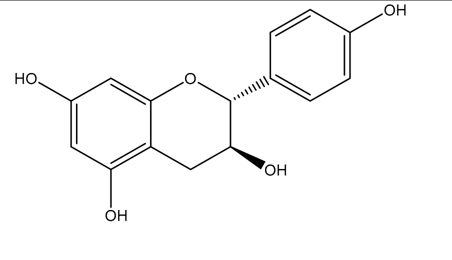
**Naringenin (2)**	**Shikoniin (3)**	**Afzelechin (4)**
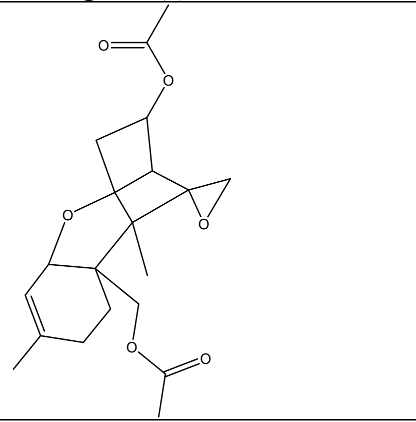	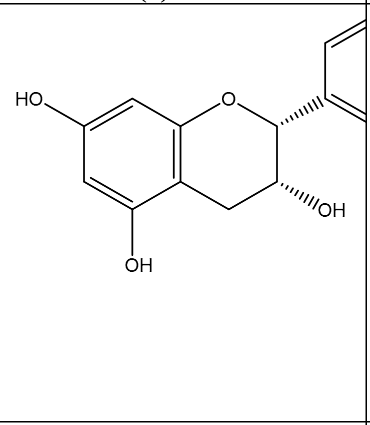	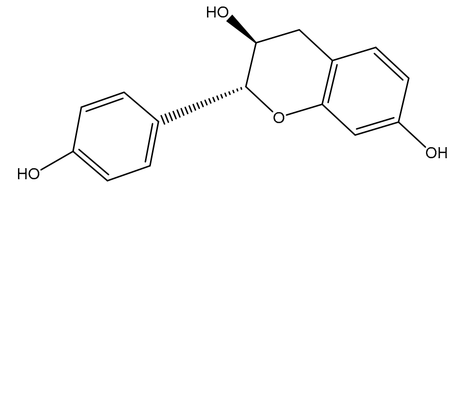
**Calonectrin (5)**	**epiAfzelechin (6)**	**Guibourtinidol (14)**
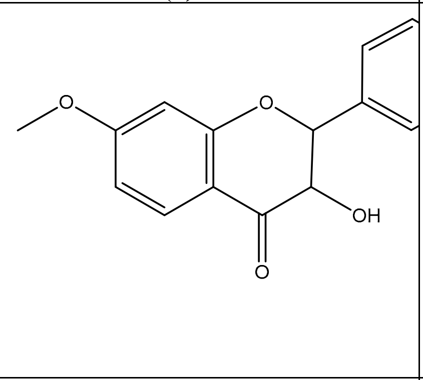	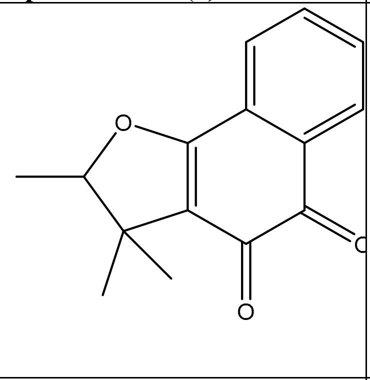	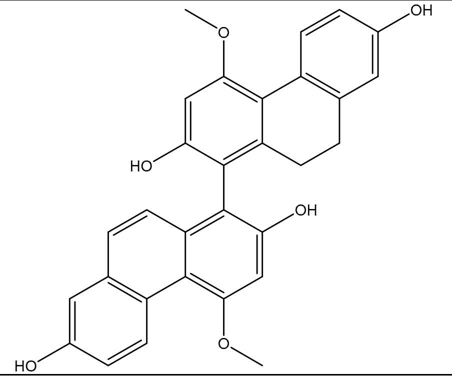
**Rhamnetin (19)**	**Dunnione (32)**	**Blestriarene (36)**

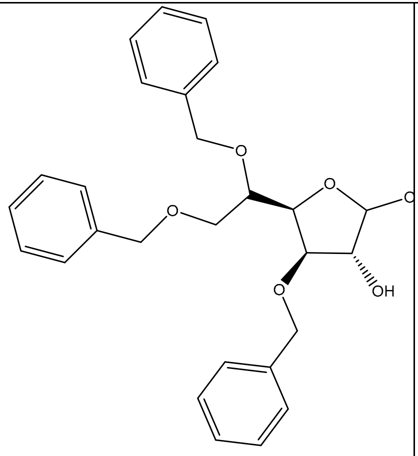

Glyvenol (38)

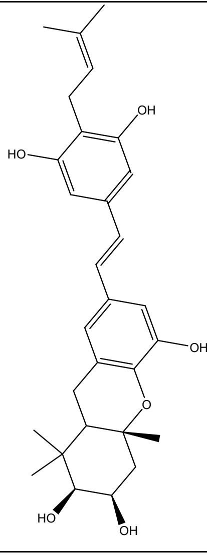

Vedelianin(40)

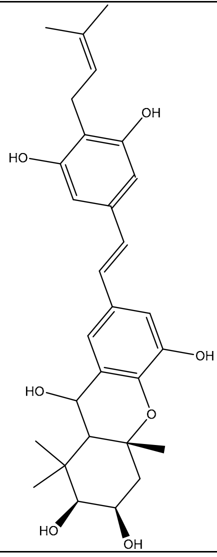

hidroxivedelianin(41)

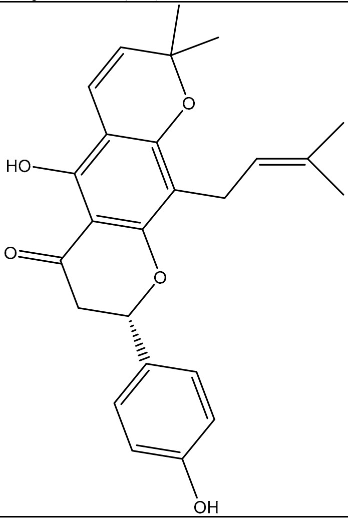

lupinifolin (46)

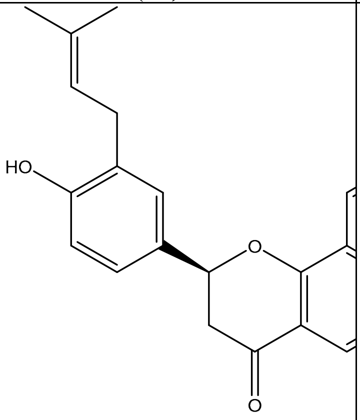

Shinflavanone (48)

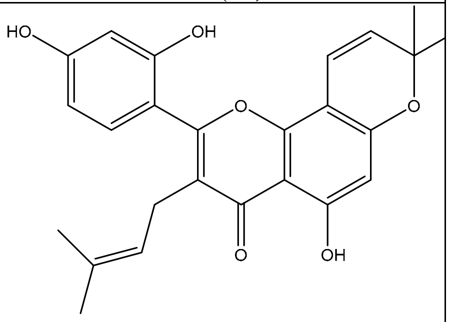

Morusin (49)

FigureS2. Structures of newly reported compounds to ZpRe.

Table S1. AntimicrobialactivityofZpRe.

**Antibacterial assay**	**MICs in μg/mL**	
	**ZpRe**	**Cefotaxime**
Staphylococcus aureus methicillin-sensitive ATCC 29213	125	0.5
Staphylococcus aureus methicillin-resistant ATCC 43300	250	0.5
Staphylococcus aureus	125	0.5

methicillin-resistant-MQ-1		
Staphylococcus aureus methicillin-resistant-MQ-2	125	0.75
Streptococcus pyogenes-1	250	1
Streptococcus agalactiae-MQ3	>250	1
Escherichia coli ATCC 25922	>250	1.9
